# Afatinib reverses multidrug resistance in ovarian cancer via dually inhibiting ATP binding cassette subfamily B member 1

**DOI:** 10.18632/oncotarget.4536

**Published:** 2015-07-20

**Authors:** Sheng-qi Wang, Shi-ting Liu, Bo-xin Zhao, Fu-heng Yang, Ya-tian Wang, Qian-ying Liang, Ya-bin Sun, Yuan Liu, Zhi-hua Song, Yun Cai, Guo-feng Li

**Affiliations:** ^1^ Department of Pharmacy, Nanfang Hospital, Southern Medical University, Guangzhou, 510515, China; ^2^ GCP Center, Nanfang Hospital, Southern Medical University, Guangzhou, 510515, China

**Keywords:** tyrosine kinase inhibitor, afatinib, ovarian cancer, multidrug resistance, ABCB1

## Abstract

ABCB1-mediated multidrug resistance (MDR) remains a major obstacle to successful chemotherapy in ovarian cancer. Herein, afatinib at nontoxic concentrations significantly reversed ABCB1-mediated MDR in ovarian cancer cells *in vitro* (*p* < 0.05). Combining paclitaxel and afatinib caused tumor regressions and tumor necrosis in A2780T xenografts *in vivo*. More interestingly, unlike reversible TKIs, afatinib had a distinctive dual-mode action. Afatinib not only inhibited the efflux function of ABCB1, but also attenuated its expression transcriptionally via down-regulation of PI3K/AKT and MAPK/p38-dependent activation of NF-κB. Furthermore, apart from a substrate binding domain, afatinib could also bind to an ATP binding domain of ABCB1 through forming hydrogen bonds with Gly533, Gly534, Lys536 and Ala560 sites. Importantly, mutations in these four binding sites of ABCB1 and the tyrosine kinase domain of EGFR were not correlated with the reversal activity of afatinib on MDR. Given that afatinib is a clinically approved drug, our results suggest combining afatinib with chemotherapeutic drugs in ovarian cancer. This study can facilitate the rediscovery of superior MDR reversal agents from molecular targeted drugs to provide a more effective and safer way of resensitizing MDR.

## INTRODUCTION

ABCB1-mediated MDR remains a major obstacle to successful chemotherapy in the clinic [1–5]. ABCB1 is highly expressed in human ovarian cancer and its overexpression is correlated inversely with a benign response to chemotherapy and good clinical prognosis [6, 7]. For this reason, tremendous efforts have been made to discover or synthesize ABCB1 inhibitors to reverse ABCB1-mediated MDR in the past decades [8–11]. One more efficient solution is to rediscover new applications of existing drugs with known pharmacokinetics and safety profiles.

Nowadays, tyrosine kinases have become the second most important group of drug targets after G-protein-coupled receptors [12]. It is reported that several TKIs could only statically inhibit the efflux function of ABCB1 and thus enhance the anticancer efficacy of chemotherapeutic drugs [13–17]. However, the mechanisms are still not very clear. Furthermore, the binding model between TKIs and ABCB1 was rarely investigated.

The most critical challenge for TKI therapeutics is also the development of drug-resistance [18]. Despite the initial response, patients almost invariably become resistant to TKIs gradually and relapse after several months. One important reason is that most approved TKIs are transported by ABC transporters, which results in an altered pharmacokinetics of these TKIs in cancer patients [18]. More seriously, as a substrate, longtime treatment often induces the up-regulation of ABC transporters, which further aggravates the resistance of conventional chemotherapy drugs [19, 20]. Therefore, it is of great medical significance that if a TKI could dynamically attenuate the expression of ABCB1, as this may further enhance the efficacy of conventional chemotherapy drugs and avoid its own drug-resistance. Unfortunately, to date, no TKIs at reversal concentrations have been reported to attenuate the expression of ABCB1.

Afatinib is the first irreversible multi-targeted TKI, functioning by selectively inhibiting EGFR and HER-2 and thereby attenuating the downstream oncogenic signaling pathways correlated with cancer proliferation, invasion, metastasis and angiogenesis. It obtained FDA approval for first-line treatment of patients with EGFR-mutated non-small cell lung cancer (NSCLC) in June 2013 and is also in clinical trials for various solid tumors with encouraging prospects [21–23]. Considering the increasing use of afatinib and the universality of ABCB1 substrate drugs in the clinic, an understanding of the interaction between afatinib and ABCB1 is necessary and of vital medical significance for dosage adjustment of ABCB1 substrate drugs during their combination treatment with afatinib. However, until now, no studies have reported the interaction between afatinib and ABCB1.

Unlike reversible TKIs, afatinib is an ATP-competitive aniline-quinazoline compound that covalently binds to the Cys773 of EGFR and Cys805 of HER-2 and irreversibly inhibits them [24, 25]. An irreversible tyrosine kinase blockade may result in the longer suppression of correlated signaling than reversible TKIs, thus producing more outstanding therapeutic effects [26, 27]. In consideration of its dual receptor and irreversible inhibition, we wonder whether afatinib is also superior in reacting with ABCB1 and whether the irreversible inhibition will offer some unique effects.

In this paper, *in vitro*, *in vivo* and *in silico* experiments were conducted to determine whether afatinib could resensitize multidrug resistant cancer cells to conventional chemotherapy drugs by interacting with ABCB1 and to uncover its mechanisms.

## RESULTS

### Afatinib reversed ABCB1-mediated MDR in ABCB1-overexpressing ovarian cancer cells *in vitro*

**Figure 1:**
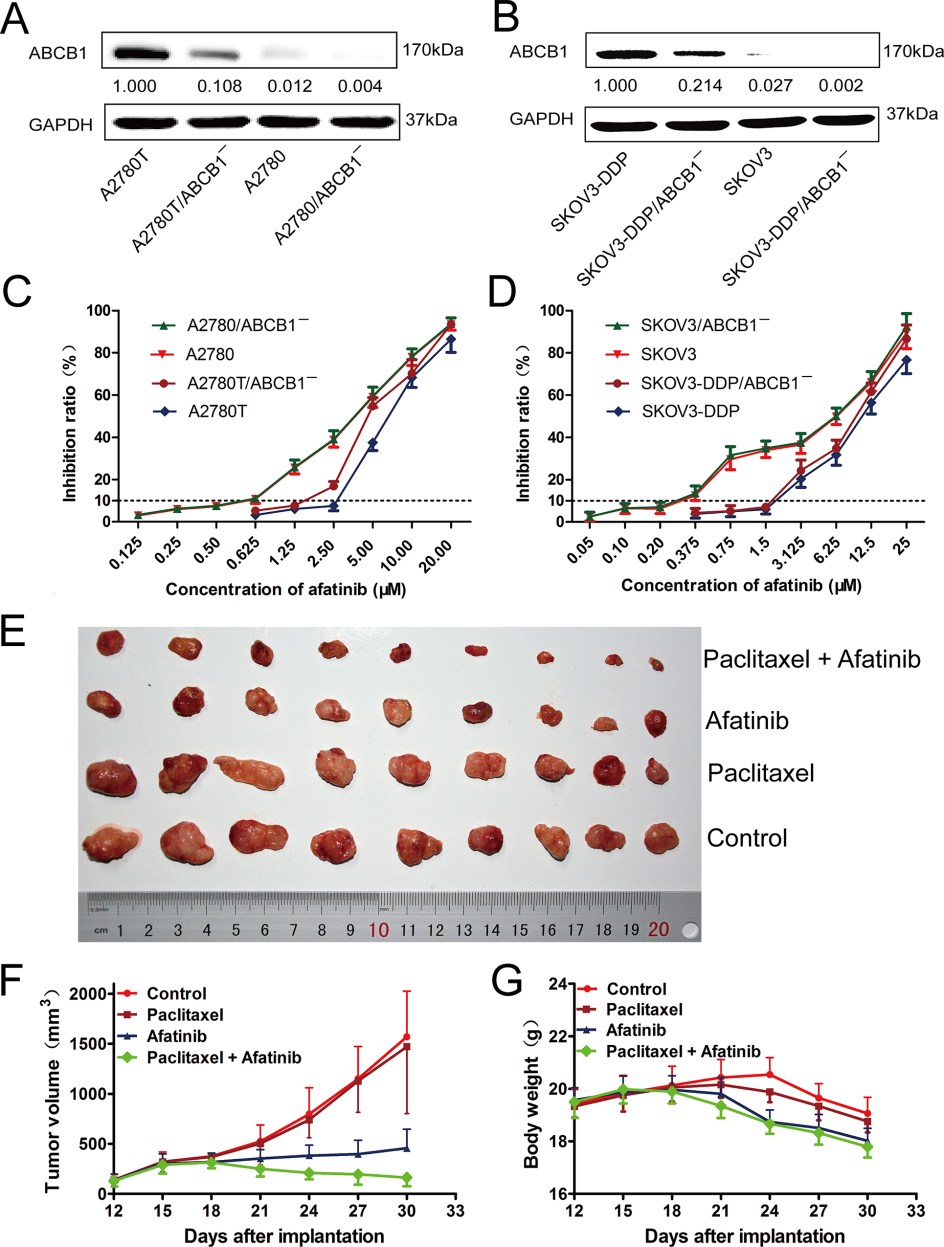
The cytotoxicity of afatinib in different kinds of human ovarian cancer cell lines *in vitro* and the reversal effect of afatinib on ABCB1-mediated MDR *in vivo* **A. and B.** The expression levels of ABCB1 protein in different kinds of ovarian cancer cell lines. **C. and D.** The growth curve of different kinds of ovarian cancer cell lines when treated with different concentrations of afatinib for 48 hours. Each point represents the mean ± SD from three independent experiments performed in triplicate. **E–G.** Afatinib reversed ABCB1-mediated MDR in A2780T xenografts *in vivo*. The various treatments were as follows: control (saline, q3d × 6, intraperitoneally, [i.p.]); paclitaxel (18 mg/kg, i.p., q3d × 6); afatinib (70 mg/kg, i.p., q3d × 6); paclitaxel (18 mg/kg, i.p., q3d × 6) plus afatinib (70 mg/kg, i.p., q3d × 6). (E) Tumor sizes of each group. (F) Variations of tumor volumes with time. (G) Variations of body weights with time.

As shown in Figure 1A and 1B, the expression levels of ABCB1 in four kinds of ovarian cancer cells (A2780T, A2780, SKOV3-DDP and SKOV3) were knocked down by ABCB1-specific siRNA. Subsequently, MTT assays were conducted to obtain the combination concentrations of afatinib in different kinds of cells. As shown in Figure 1C and 1D, there was a significant difference in the susceptibility of various cells to afatinib alone. More than 90% of the cells were viable with a concentration up to 0.5 μM afatinib for A2780 cells, 2.5 μM for A2780T cells, 0.5 μM for A2780/ABCB1¯ cells and 1.25 μM for A2780T/ABCB1¯ cells. Accordingly, afatinib at concentrations of 0.5 μM, 2.5 μM, 0.5 μM and 1.25 μM were used as the maximum concentrations in the reversal assays for A2780, A2780T, A2780/ABCB1¯ and A2780T/ABCB1¯ cells, respectively. Similarly, afatinib at concentrations of 0.2 μM, 1.5 μM, 0.2 μM and 1.5 μM were used as the maximum concentrations in the reversal assays for SKOV3, SKOV3-DDP, SKOV3/ABCB1¯ and SKOV3-DDP/ABCB1¯ cells, respectively.

As shown in Table 1, The ABCB1-overexpressing A2780T and SKOV3-DDP cells showed significant resistant phenotype to ABCB1 substrate drugs (paclitaxel and adriamycin). Afatinib at nontoxic concentrations strongly decreased the IC_50_ values of paclitaxel and adriamycin in a concentration-dependent manner in ABCB1-overexpressing A2780T and SKOV3-DDP cells. In contrast, afatinib didn't alter the cytotoxicity of paclitaxel and adriamycin in A2780 and SKOV3 cells, which expressed low levels of ABCB1 protein. As shown in Table 2, knockdown of ABCB1 in A2780T and SKOV3-DDP cells further enhanced the reversal effect of afatinib on MDR. Taken together, these results indicated that afatinib could reverse the MDR to ABCB1 substrate drugs in ABCB1-overexpressing ovarian cancer cells. In addition, afatinib could also reverse the MDR to ABCG2 substrate drugs (mitoxantrone and topotecan) in A2780T and SKOV3-DDP cells, while having no effect on the MDR to chlorambucil, which was an ABCC1 substrate drug (data not shown).

**Table 1:** Afatinib reversed ABCB1-mediated MDR *in vitro*

Compounds	IC_50_ ± SD (μM; fold-reversal factor)			
	A2780		A2780T	
Paclitaxel		0.039 ± 0.003 (1.00)		198.92 ± 14.85 (1.00)
	+0.125 μM afatinib	0.039 ± 0.002 (1.00)	+0.625 μM afatinib	115.53 ± 6.65* (1.72)
	+0.25 μM afatinib	0.038 ± 0.002 (1.03)	+1.25 μM afatinib	19.74 ± 1.19* (10.08)
	+0.5 μM afatinib	0.039 ± 0.002 (1.00)	+2.5 μM afatinib	8.86 ± 0.72* (22.45)
	+0.5 μM lapatinib	0.039 ± 0.002 (1.00)	+2.5 μM lapatinib	90.14 ± 8.14* (2.21)
	+10 μM verapamil	0.038 ± 0.003 (1.03)	+10 μM verapamil	50.27 ± 3.38* (3.96)
Adriamycin		1.17 ± 0.06 (1.00)		27.69 ± 2.11 (1.00)
	+0.125 μM afatinib	1.16 ± 0.09 (1.01)	+0.625 μM afatinib	6.24 ± 0.37* (4.44)
	+0.25 μM afatinib	1.15 ± 0.07 (1.02)	+1.25 μM afatinib	3.93 ± 0.22* (7.05)
	+0.5 μM afatinib	1.15 ± 0.08 (1.02)	+2.5 μM afatinib	1.57 ± 0.13* (17.64)
	+0.5 μM lapatinib	1.16 ± 0.08 (1.01)	+2.5 μM lapatinib	5.31 ± 0.44* (5.21)
	+10 μM verapamil	1.18 ± 0.07 (0.99)	+10 μM verapamil	12.87 ± 0.54* (2.15)
Cisplatinum		23.19 ± 1.54 (1.00)		24.82 ± 1.37 (1.00)
	+0.125 μM afatinib	23.03 ± 1.62 (1.01)	+0.625 μM afatinib	16.67 ± 0.97* (1.49)
	+0.25 μM afatinib	22.97 ± 1.33 (1.01)	+1.25 μM afatinib	14.81 ± 0.65* (1.68)
	+0.5 μM afatinib	22.81 ± 1.47 (1.02)	+2.5 μM afatinib	8.81 ± 0.73* (2.82)
	SKOV3		SKOV3-DDP	
Paclitaxel		0.033 ± 0.002 (1.00)		41.79 ± 3.67 (1.00)
	+0.05 μM afatinib	0.033 ± 0.002 (1.00)	+0.375 μM afatinib	23.53 ± 2.47* (1.78)
	+0.10 μM afatinib	0.033 ± 0.003 (1.00)	+0.75 μM afatinib	9.82 ± 0.80* (4.26)
	+0.2 μM afatinib	0.032 ± 0.002 (1.03)	+1.5 μM afatinib	5.75 ± 0.47* (7.27)
	+0.2 μM lapatinib	0.033 ± 0.002 (1.00)	+1.5 μM lapatinib	22.46 ± 1.45* (1.86)
	+10 μM verapamil	0.032 ± 0.003 (1.03)	+10 μM verapamil	21.54 ± 1.69* (1.94)
Adriamycin		0.97 ± 0.07 (1.00)		18.13 ± 1.54 (1.00)
	+0.05 μM afatinib	0.97 ± 0.08 (1.00)	+0.375 μM afatinib	9.17 ± 1.05* (1.98)
	+0.1 μM afatinib	0.96 ± 0.07 (1.01)	+0.75 μM afatinib	3.58 ± 0.29* (5.06)
	+0.2 μM afatinib	0.96 ± 0.06 (1.01)	+1.5 μM afatinib	1.25 ± 0.13* (14.50)
	+0.2 μM lapatinib	0.95 ± 0.08 (1.02)	+1.5 μM lapatinib	8.32 ± 0.62* (2.18)
	+10 μM verapamil	0.95 ± 0.06 (1.02)	+10 μM verapamil	9.29 ± 0.72* (1.95)
Cisplatinum		20.68 ± 1.85 (1.00)		47.31 ± 3.58 (1.00)
	+0.05 μM afatinib	20.53 ± 1.21 (1.01)	+0.375 μM afatinib	38.59 ± 3.26* (1.23)
	+0.1 μM afatinib	20.37 ± 1.09 (1.02)	+0.75 μM afatinib	29.43 ± 2.35* (1.61)
	+0.2 μM afatinib	20.21 ± 1.35 (1.02)	+1.5 μM afatinib	17.92 ± 1.30* (2.64)

Note: Cell survival was determined by MTT assays as described in Materials and Methods. Data are the mean ± SD of at least three independent experiments performed in triplicate. The fold-reversal factor of MDR (values given in parentheses) was calculated by dividing the IC_50_ for cells with the anticancer drugs in the absence of reversal agents by that obtained in the presence of reversal agents.

* *P* < 0.01, versus the values obtained in the absence of reversal agents.

**Table 2:** Afatinib reversed ABCB1-mediated MDR in transfected cells *in vitro*

Compounds	IC_50_ ± SD (μM; fold-reversal factor)			
	A2780/ABCB1¯		A2780T/ABCB1¯	
Paclitaxel		0.035 ± 0.002 (1.00)		126.23 ± 9.31 (1.00)
	+0.125 μM afatinib	0.035 ± 0.002 (1.00)	+0.625 μM afatinib	84.38 ± 7.51* (1.50)
	+0.25 μM afatinib	0.034 ± 0.002 (1.03)	+1.25 μM afatinib	13.62 ± 0.96* (9.27)
	+0.5 μM afatinib	0.035 ± 0.003 (1.00)	+2.5 μM afatinib	6.55 ± 0.41* (19.27)
	+10 μM verapamil	0.034 ± 0.002(1.03)	+10 μM verapamil	40.69 ± 2.73* (3.10)
Adriamycin		1.09 ± 0.07 (1.00)		19.40 ± 1.47 (1.00)
	+0.125 μM afatinib	1.07 ± 0.05 (1.02)	+0.625 μM afatinib	4.55 ± 0.19* (4.26)
	+0.25 μM afatinib	1.09 ± 0.06 (1.00)	+1.25 μM afatinib	1.87 ± 0.15* (10.37)
	+0.5 μM afatinib	1.09 ± 0.09(1.00)	+2.5 μM afatinib	1.53 ± 0.13* (12.68)
	+10 μM verapamil	1.08 ± 0.04 (1.01)	+10 μM verapamil	9.63 ± 0.49* (2.01)
Cisplatinum		22.55 ± 1.38 (1.00)		23.74 ± 0.00 (1.00)
	+0.125 μM afatinib	22.36 ± 0.93 (1.01)	+0.625 μM afatinib	16.38 ± 1.13* (1.45)
	+0.25 μM afatinib	22.39 ± 1.48 (1.01)	+1.25 μM afatinib	14.25 ± 0.85* (1.67)
	+0.5 μM afatinib	22.46 ± 1.31 (1.00)	+2.5 μM afatinib	7.72 ± 0.49* (3.08)
	SKOV3/ABCB1¯		SKOV3-DDP/ABCB1¯	
Paclitaxel		0.029 ± 0.002 (1.00)		28.67 ± 3.01 (1.00)
	+0.05 μM afatinib	0.029 ± 0.002 (1.00)	+0.375 μM afatinib	19.45 ± 1.28* (1.47)
	+0.1 μM afatinib	0.028 ± 0.003 (1.03)	+0.75 μM afatinib	7.51 ± 0.39* (3.82)
	+0.2 μM afatinib	0.029 ± 0.002 (1.00)	+1.5 μM afatinib	3.04 ± 0.25* (9.43)
	+10 μM verapamil	0.028 ± 0.002(1.03)	+10 μM verapamil	16.71 ± 1.29* (1.72)
Adriamycin		0.96 ± 0.05 (1.00)		13.26 ± 1.15 (1.00)
	+0.05 μM afatinib	0.96 ± 0.05 (1.00)	+0.375 μM afatinib	8.63 ± 0.57* (1.54)
	+0.1 μM afatinib	0.95 ± 0.07 (1.01)	+0.75 μM afatinib	2.17 ± 0.15* (6.11)
	+0.2 μM afatinib	0.96 ± 0.06(1.00)	+1.5 μM afatinib	1.06 ± 0.09* (12.51)
	+10 μM verapamil	0.96 ± 0.05 (1.00)	+10 μM verapamil	7.91 ± 0.34* (1.68)
Cisplatinum		20.17 ± 1.51 (1.00)		45.72 ± 3.61 (1.00)
	+0.05 μM afatinib	20.14 ± 1.73 (1.00)	+0.375 μM afatinib	38.35 ± 2.74* (1.19)
	+0.1 μM afatinib	19.89 ± 1.47 (1.01)	+0.75 μM afatinib	29.07 ± 2.15* (1.57)
	+0.2 μM afatinib	19.62 ± 1.25 (1.03)	+1.5 μM afatinib	16.91 ± 1.38* (2.70)

Note: Cell survival was determined by MTT assays as described in Materials and Methods. Data are the mean ± SD of at least three independent experiments performed in triplicate. The fold-reversal factor of MDR (values given in parentheses) was calculated by dividing the IC_50_ for cells with the anticancer drugs in the absence of reversal agents by that obtained in the presence of reversal agents.

* *P* < 0.01, versus the values obtained in the absence of reversal agents.

### Afatinib reversed ABCB1-mediated MDR *in vivo*

As shown in Supplementary Figure S1B, the average weights of tumors were 0.707 ± 0.229 g, 0.655 ± 0.289 g, 0.247 ± 0.088 g and 0.113 ± 0.079 g for control, paclitaxel, afatinib and the combination group, respectively. There were no significant differences in both the tumor volumes (Figure 1E and 1F, Supplementary Figure S1A) and tumor weights (Supplementary Figure S1B) between paclitaxel group and control group (*p* > 0.05), indicating the *in vivo* resistance to paclitaxel. However, the combination of paclitaxel and afatinib not only significantly delayed the growth of A2780T xenografts, but also induced significant tumor regressions with an inhibition rate of 84.02% (Figure 1F). Furthermore, compared with afatinib group, no treatment-correlated mortality or apparent decrease in body weight (Figure 1G) were observed, indicating the combination didn't induce additional adverse drug reactions.

### Afatinib enhanced the paclitaxel-induced apoptosis *in vitro* and *in vivo*

**Figure 2:**
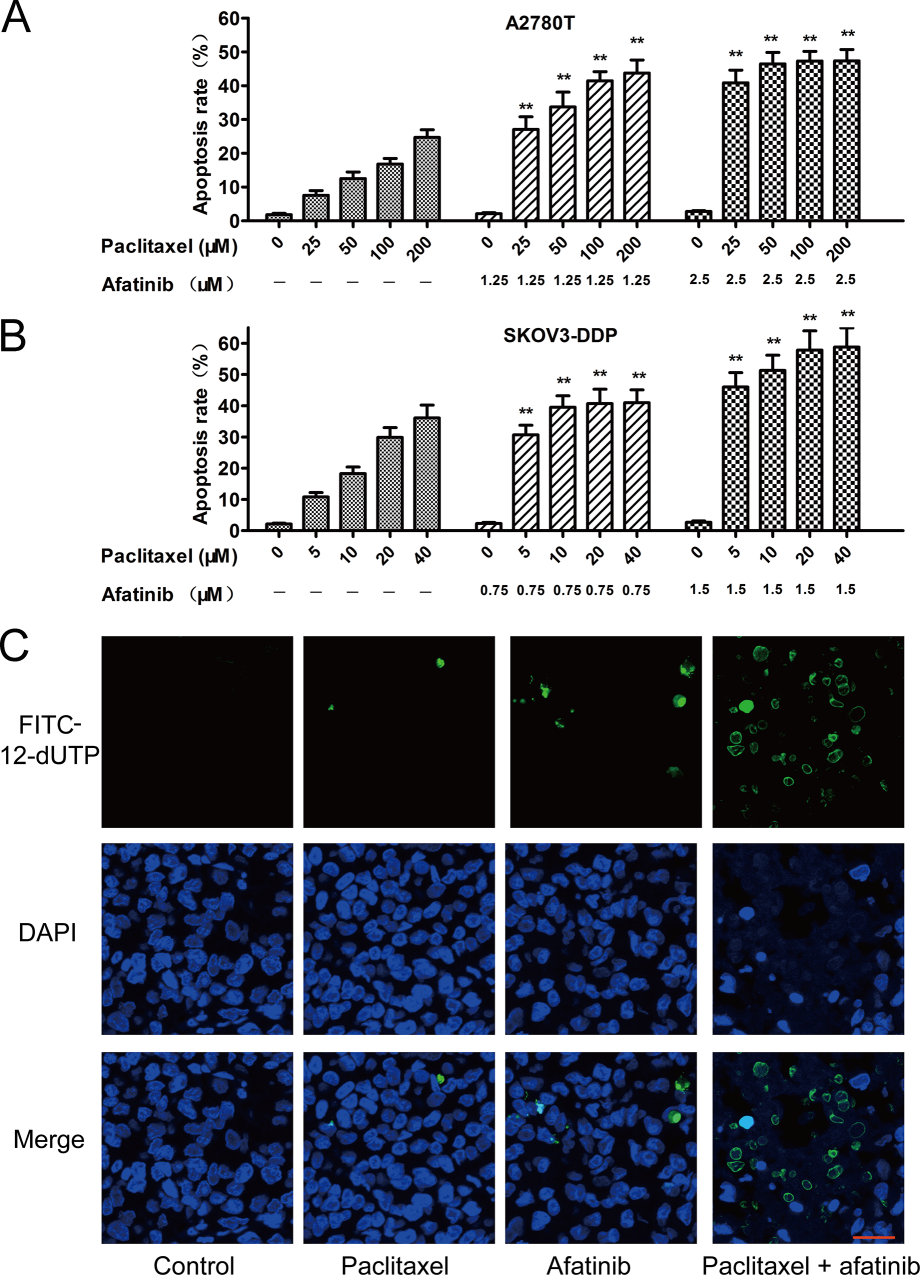
Afatinib enhanced the paclitaxel-induced apoptosis *in vitro* and *in vivo* **A. and B.** Afatinib enhanced the paclitaxel-induced apoptosis *in vitro*. Cells were treated with indicated concentrations of paclitaxel in the absence or presence of indicated concentrations of afatinib for 48 hours. Data are represented as the mean ± SD from three independent experiments performed in triplicate. **P < 0.01 versus the group treated with the same concentrations of paclitaxel in the absence of afatinib. **C.** Effects of afatinib on paclitaxel-induced apoptosis in tumor tissues were investigated by the Tunnel assay. Apoptotic cells were stained with FITC-12-dUTP (green). Cell nucleus were stained with DAPI (blue). Scale bar = 20 μM.

As shown in Figure 2A and 2B, afatinib at nontoxic concentrations significantly increased the percentage of apoptosis induced by paclitaxel in multidrug-resistant A2780T and SKOV3-DDP cells. Furthermore, few isolated positive nuclei were observed in tumors of saline, paclitaxel or afatinib group, whereas abundant apoptotic nuclei were clearly exhibited in the combination group (Figure 2C). Moreover, combining paclitaxel with afatinib induced remarkable nucleus rupture and nuclear mass leak, indicating a high occurrence ratio of tumornecrosis.

### Afatinib inhibited the efflux function of ABCB1

**Figure 3:**
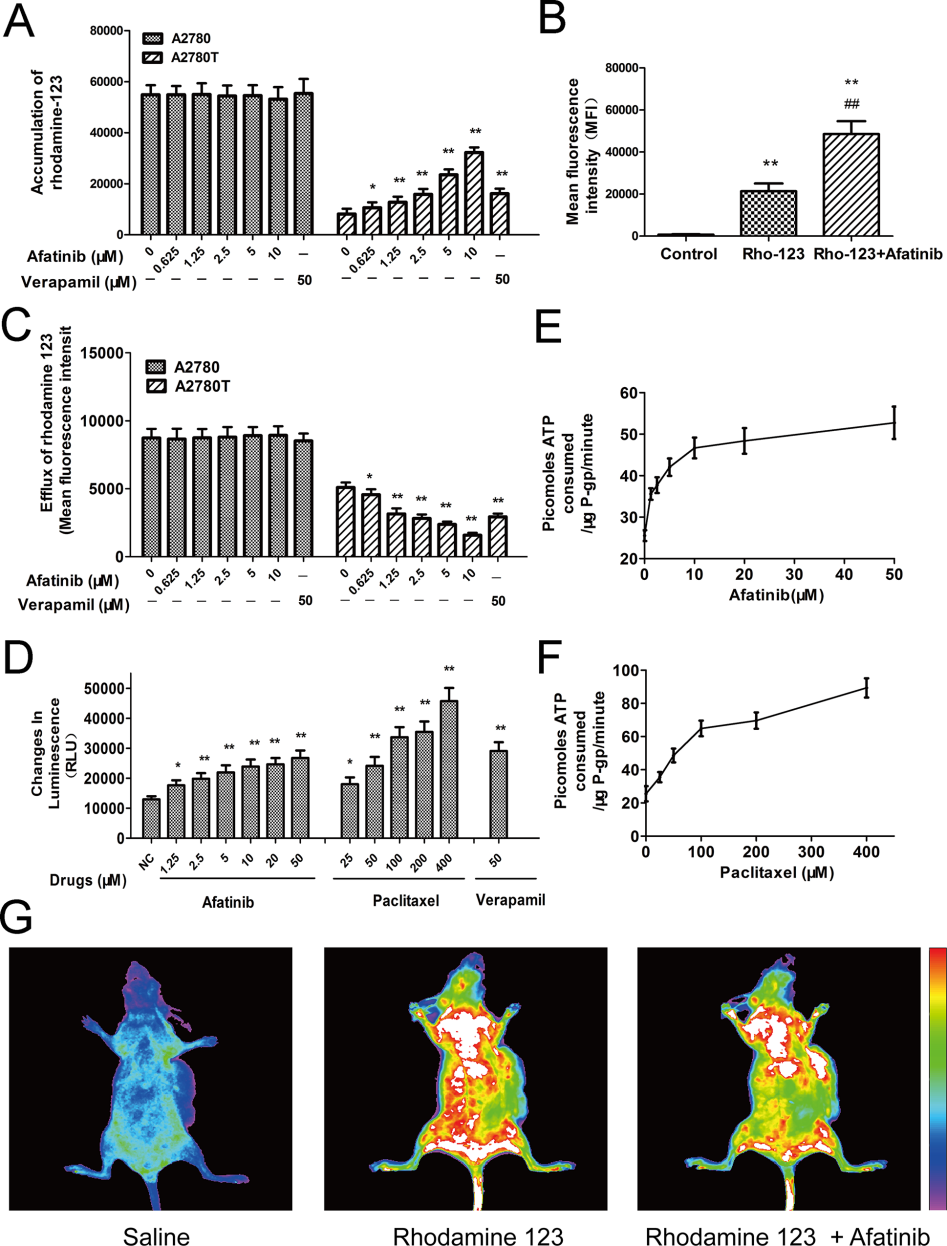
Afatinib inhibited the efflux function and stimulated the ATPase activity of ABCB1 **A.** Effects of afatinib on the intracellular accumulation of rhodamine 123 in A2780 and A2780T cells. **B.** Effects of afatinib on the accumulation of rhodamine 123 in A2780T xenografts. Figure 3B is the quantitation of the *in vivo* fluorescence shown in Figure 3G. **C.** Effects of afatinib on the efflux of rhodamine 123 in A2780 and A2780T cells. **D.** Effects of afatinib, paclitaxel and verapamil on the ATPase activity of ABCB1. **E. and F.** Afatinib and paclitaxel increased the consumption speed of ATP in recombinant human ABCB1 membranes. **G.** Effects of afatinib on the accumulation of rhodamine 123 in A2780T xenografts. Data are represented as the mean ± SD from three independent experiments performed in triplicate. *p < 0.05 vs control group; **p < 0.01 vs control group; ^##^*p* < 0.01 vs Rho-123 group.

As shown in Figure 3A, afatinib remarkably increased the intracellular accumulation of rhodamine 123 (a fluorescent substrate of ABCB1) in ABCB1-overexpressing A2780T cells, while having no effect on that in A2780 cells. More meaningfully, afatinib also significantly increased the accumulation of rhodamine 123 in A2780T xenografts by 2.28 folds (Figure 3B, 3G). Since ABCB1 was an efflux pump, the *in vitro* transport assay was conducted to examine whether the increase of accumulation was achieved by decreasing the efflux function of ABCB1. As shown in Figure 3C, afatinib significantly decreased the efflux of rhodamine 123 in A2780T cells while having no effect on that in A2780 cells. To sum up, afatinib significantly increased the accumulation of rhodamine 123 both *in vitro* and *in vivo* by inhibiting the efflux function of ABCB1.

### Afatinib stimulated the ATPase activity of ABCB1

Energy consumption during the efflux process of ABCB1 comes from ATP hydrolysis. Therefore, effect of afatinib on ABCB1-mediated ATP hydrolysis was evaluated. Both afatinib and paclitaxel stimulated the ATPase activity of ABCB1 (Figure 3D) during a short-time incubation with recombinant human ABCB1 membranes. Generally, the substrates of ABCB1 stimulate its ATPase activity. Hence, like paclitaxel, afatinib may also be a substrate of ABCB1. Besides, the concentrations required for 50% stimulation of the ATPase activity of ABCB1 were about 2.5 μM for afatinib and 70.1 μM for paclitaxel, suggesting that afatinib had much stronger affinity with ABCB1 than paclitaxel (Figure 3E, 3F).

### Afatinib attenuated the expression of ABCB1by inhibiting the activation of NF-κB

**Figure 4:**
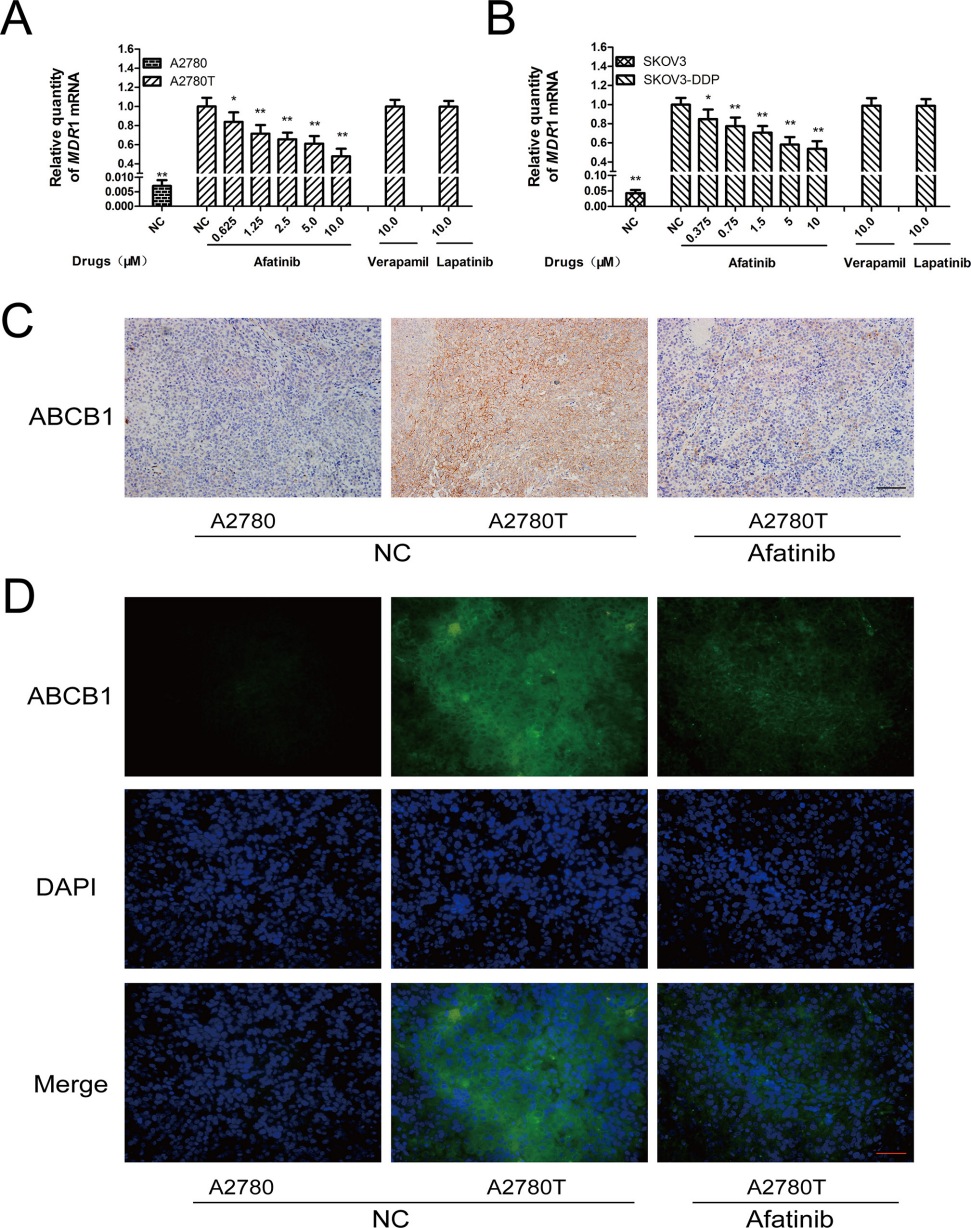
Afatinib attenuated the expression of ABCB1 *in vitro* and *in vivo* **A. and B.** Effects of afatinib on the expression of *MDR*1 mRNA in ovarian cancer cells. Shown are the mean relative mRNA expression level normalized to untreated cells from three independent experiments performed in triplicate with standard deviations. *p < 0.05 vs control group of multidrug-resistant cells; **p < 0.01 vs control group of multidrug-resistant cells. **C.** Effects of afatinib on the expression of ABCB1 protein in tumor tissues were detected by immunohistochemistry. Scale bar = 100 μM. **D.** Effects of afatinib on the protein expression of ABCB1 in tumor tissues were detected by immunofluorescence. Scale bar = 50 μM.

**Figure 5:**
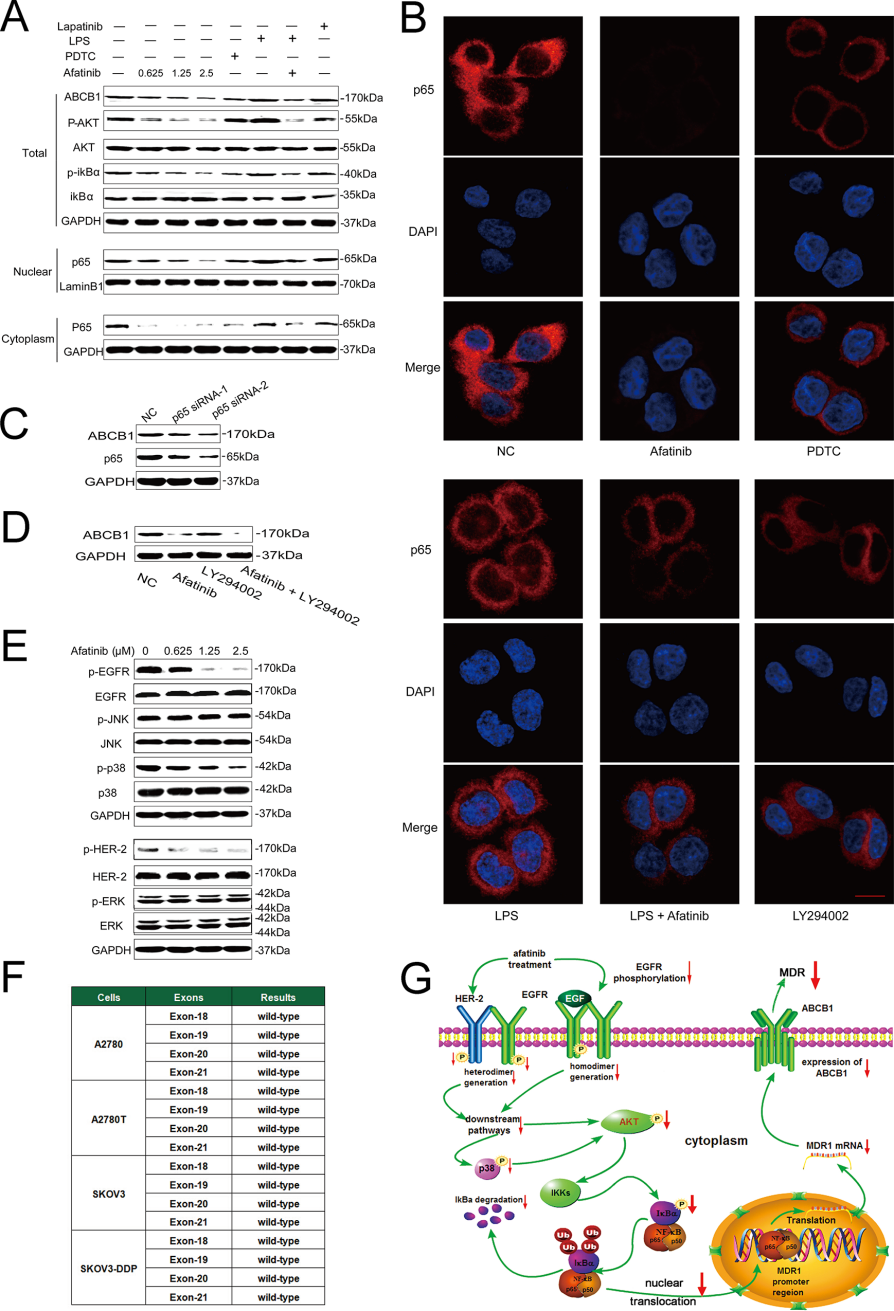
Afatinib attenuated the expression of ABCB1 by inhibiting its transcription via down-regulation of PI3K/AKT and MAPK/p38-dependent activation of NF-κB **A.** Effects of afatinib on the expression of correlated proteins. A2780T cells were treated with 0.625–2.5 μM afatinib for 48 hours, or 10 μM PDTC for 2 hours, or 1 μg/ml LPS for 2 hours, or 2.5 μM lapatinib for 48 hours, or a combination treatment of 1 μg/ml LPS for 2 hours followed by an incubation with 2.5 μM afatinib for 48 hours, respectively. **B.** Effects of different treatments on the nuclear translocation of the NF-κB subunit p65. A2780T cells were treated with 2.5 μM afatinib for 48 hours, or 10 μM PDTC for 2 hours, or 1 μg/ml LPS for 2 hours, or 10 μM LY294002 for 2 hours, or a combination treatment of 1 μg/ml LPS pretreatment for 2 hours followed by an incubation with 2.5 μM afatinib for 48 hours, respectively. Subsequently, the NF-κB subunit p65 (red) was localized by immunofluorescence and a confocal microscopy. Nuclei were stained with DAPI (blue). Scale bar = 10 μM. **C.** p65 specific siRNA attenuated the expression of ABCB1 protein in A2780T cells. **D.** A combination treatment of afatinib and LY294002 induced additive inhibitory effects on ABCB1 expression in A2780T cells. A2780T cells were treated with 2.5 μM afatinib for 48 hours, or 10 μM LY294002 for 2 hours, or a combination treatment of 2.5 μM afatinib for 48 hours followed by an incubation with 10 μM LY294002 for 2 hours. **E.** Effects of afatinib on the MAPK pathway. A2780T cells were treated with indicated concentrations of afatinib for 48 hours. **F.** No mutations occurred in exon 18, exon 19, exon 20 and exon 21 regions of EGFR gene in four kinds of ovarian cancer cell lines. **G.** A schematic diagram of the mechanism by which afatinib attenuated the expression of ABCB1.

Afatinib could dramatically attenuate the expression of *MDR*1 mRNA (Figure 4A and 4B) and ABCB1 protein (Figure 5A, Supplementary Figure S2) in ABCB1-overexpressing A2780T and SKOV3-DDP cells in a concentration-dependent manner, whereas lapatinib (a reversible TKI targeting to EGFR and HER-2) having no effect on that. Moreover, both immunohistochemical (Figure 4C) and immunofluorescent assays (Figure 4D) showed that afatinib could also attenuate the expression of ABCB1 protein in tumor tissues *in vivo*. In addition, both PDTC (a specific NF-κB inhibitor) and p65-specific siRNA could attenuate the expression of ABCB1 protein (Figure 5A and 5C) and *MDR*1 mRNA (Supplementary Figure S3). To evaluate whether NF-κB pathway was involved in the observed suppression of ABCB1 expression by afatinib, the effect of afatinib on the NF-κB pathway was assessed. As shown in Figure 5A, afatinib attenuated the expression of the NF-κB subunit p65 both in the nucleus and the cytoplasm. Meanwhile, afatinib decreased the phosphorylation and degradation of IκBα. The nuclear translocation of p65 is essential for the activation of NF-κB pathway. As shown in Figure 5B, like PDTC, afatinib abrogated both the constitutive and LPS-induced nuclear translocation of p65. In conclusion, afatinib could attenuate the expression of ABCB1 by inhibiting the activation of the NF-κB.

PI3K/Akt pathway is closely involved in the regulation of NF-κB activity [28]. As shown in Figure 5A, afatinib remarkably blocked the phosphorylation of Akt without affecting total Akt levels, which was in consistent with Chao's study [29]. Meanwhile, LY294002, a specific inhibitor of PI3K/Akt pathway, also significantly suppressed the nuclear translocation of p65 (Figure 5B). Moreover, the combination of afatinib and LY294002 induced additive inhibitory effects on ABCB1 expression (Figure 5D), suggesting that afatinib might suppress NF-κB activity through inhibiting the PI3K/Akt pathway.

### EGFR mutations were not correlated with the reversal efficacy of afatinib on MDR in ovarian cancer

More than 90% of EGFR mutations occur in the tyrosine kinase domain, which are encoded by exon 18, exon 19, exon 20 and exon 21 of the EGFR gene [30, 31]. As shown in Figure 5F, all the four exons were wide-type in both drug-sensitive cells (A2780 and SKOV3 cells) and multidrug-resistant cells (A2780T and SKOV3-DDP cells). These results indicated that EGFR mutations were not correlated with the reversal efficacy of afatinib on MDR in ovarian cancer.

### Blockage of the MAPK/p38 pathway contributed to the reversal effect of afatinib

Inhibition of the MAPK pathway may partially restore the sensitivity to chemotherapeutic drugs in cancer cells [32]. As afatinib is a TKI targeting EGFR and HER-2, of which the major downstream signaling pathways include the Ras/Raf/MAPK pathway. Therefore, the effects of afatinib on the expression of total and phosphorylated ERK1/2, JNK and p38 were examined. As shown in Figure 5E, afatinib at reversal concentrations significantly blocked the phosphorylation of p38 in A2780T cells, whereas it didn't alter the expression level of total and phosphorylated ERK1/2 or JNK. Since MAPK/p38 is also the upstream regulatory protein of the PI3K/AKT and NF-κB pathway, these results indicated that afatinib could attenuate the expression of ABCB1 via down-regulation of PI3K/AKT and MAPK/p38-dependent activation of NF-κB (Figure 5G).

### Structural basis of afatinib as an ABCB1 inhibitor

**Figure 6:**
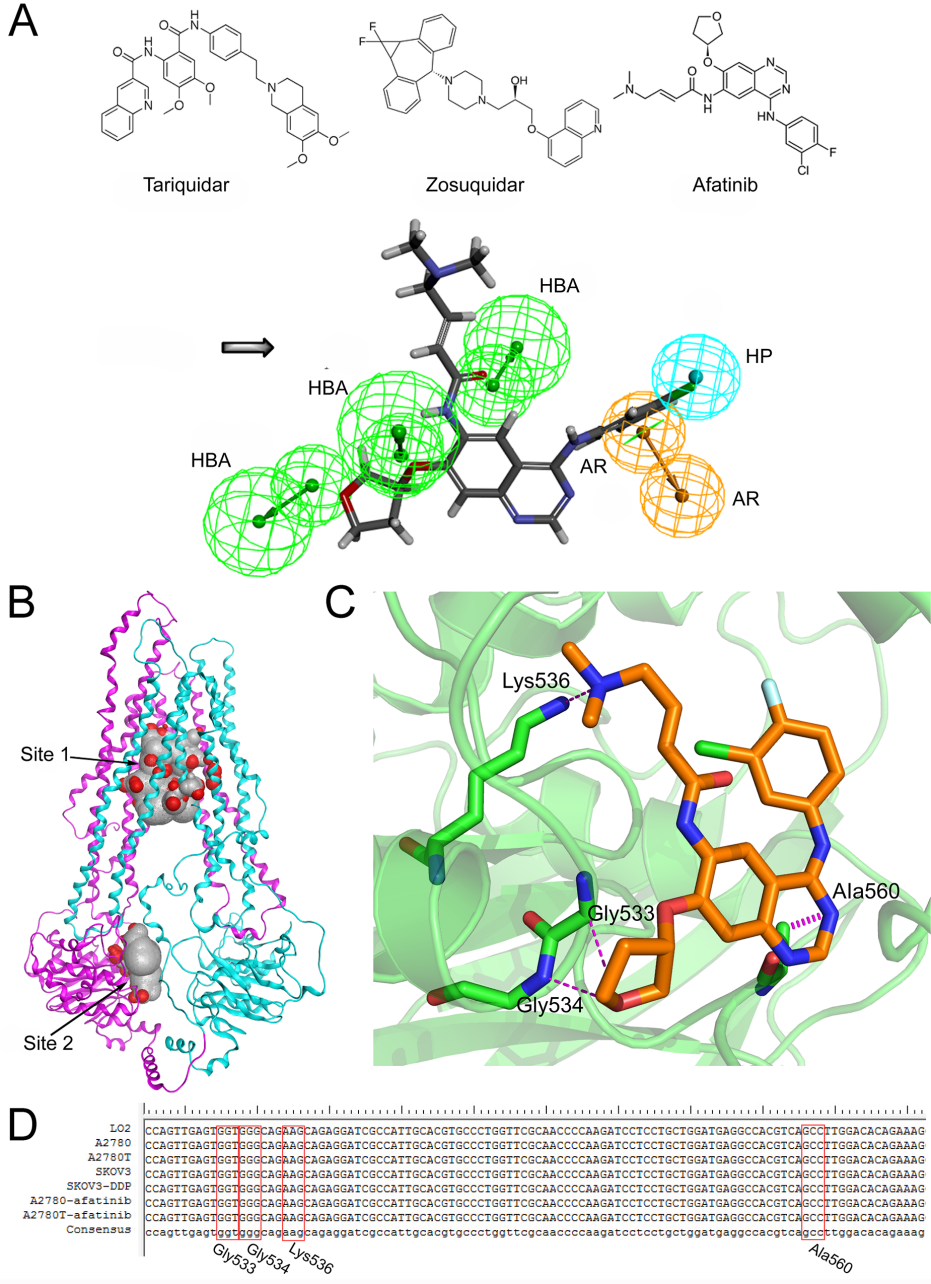
The structural basis of afatinib as an ABCB1 inhibitor and the possible binding mode between afatinib and ABCB1 **A.** The chemical structures of tariquidar, zosuquidar and afatinib (above). The generated pharmacophore model of ABCB1 inhibitors (below). AR: aromatic ring; HBA: hydrogen bond acceptor; HP: hydrophobic center. **B.** The homology model of human ABCB1protein. The substrate binding domain (**site 1**) and the ATP binding domain (**site 2**). **C.** The three dimensional representation of the binding mode between afatinib and ABCB1. **D.** Partial sequencing results of the 378-bp fragment of the *MDR*1 gene encompassing the sequence for Gly533, Gly534, Lys536 and Ala560 of ABCB1 protein.

To identify the structural basis of afatinib as an inhibitor of ABCB1, a novel pharmacophore model was developed on the basis of two selective and potent ABCB1 inhibitors including tariquidar [33] and zosuquidar [34]. The critical pharmacophore features necessary for ABCB1 inhibitors were identified (Figure 6A). The best pharmacophore model had five features, including one aromatic center (AR), one hydrophobic center (HP) and three hydrogen bond acceptors (HBAs). Afatinib formed an impressive alignment with this model. To be specific: the aromatic ring was mapped onto the benzene ring of afatinib; the hydrophobic center was mapped onto the fluorine atoms and the connected benzene ring of afatinib; and three HBAs were mapped onto the three oxygen atoms of afatinib. These results further verified the superior inhibitory activity of afatinib on ABCB1.

### Binding mode between afatinib and ABCB1

To determine the binding mode between afatinib and human ABCB1, the 3D structure of human ABCB1 (Figure 6B) was generated by homology modeling. All the scores of various validation methods including PROCHECK, ERRAT and PROSA (Supplementary Table S1), and the ramachandran plot results (Supplementary Figure S4) verified its quality. Afatinib was successfully docked into both a substrate binding domain and an ATP binding domain of this model (Figure 6B). The substrate binding domain was a large flexible binding pocket, which could host different substrates [35]. This finding also was in consistent with the universality of ABCB1 substrate drugs. Furthermore, the docking score for the ATP binding domain (−14.05 kcal/mol) was very close to that of the substrate binding domain (−14.09 kcal/mol), indicating that the binding of afatinib at the ATP binding domain might also be with high affinity. The interactions between afatinib and the ATP binding domain of ABCB1 included three hydrogen bonds and an arene-hydrogen interaction. The oxygen atom of oxolane in afatinib formed two hydrogen bonds with the backbone of Gly533 and Gly534. Interestingly, the oxygen atom of oxolane in afatinib was also identified as an HBA feature in the pharmacophore model as mentioned above, which verified the reliability of the pharmacophore model. Besides, the tertiary amine of afatinib formed a hydrogen bond with the side chain of Lys536 while the N-containing heterocyclic of afatinib formed an arene hydrogen interaction with Ala560. A 378-bp fragment of *MDR*1 gene, encompassing the sequence for Gly533, Gly534, Lys536 and Ala560 of ABCB1 protein, was amplified. As shown in Figure 6D, DNA sequencing results showed that the normal human cells (liver LO2 cells) and four kinds of ovarian cells were all wide-type in this fragment region of *MDR*1 gene. In addition, afatinib treatment also didn't induce gene mutations in these four sites in both drug-sensitive A2780 cells and multidrug resistant A2780T cells. These results indicated that mutations in these four sites were not correlated with the reversal efficacy of afatinib on MDR in multidrug resistant ovarian cancer cells. In summary, the docking simulation study indicated that afatinib might bind to the ATP binding domain of ABCB1 through forming hydrogen bonds with Gly533, Gly534, Lys536 and Ala560 sites of ABCB1.

## DISCUSSION

Multidrug resistance (MDR), mainly mediated by the ABCB1 transporter, remains a major obstacle to successful chemotherapy. Tremendous attempts have been made to circumvent or reverse ABCB1-mediated MDR. On one hand, ABCB1-mediated MDR could be indirectly overcome by exploring new drug combination strategies through exploiting the interaction between drugs and ABC transporters, which could even have extra benefits. For example, MV Blagosklonny reported that a “two drug” strategy enabled selective killing of resistant leukemia cells, whereas parental cells were fully protected [36]. In addition, it had been reported that treatment with inhibitors of caspases, that were substrates of drug transporters, could selectively permit chemotherapy-induced apoptosis in multidrug-resistant cells but protect normal cells [37]. On the other hand, discovering or synthesizing ABCB1 inhibitors to reverse ABCB1-mediated MDR is more direct and has been the primary means in the past decades [8–11].

Ovarian cancer is the most fatal malignancy of the female reproductive tract, causing more deaths than all other gynecologic cancers combined [38]. Paclitaxel and platinum combination chemotherapy has remained the global first-line chemotherapy regimen for patients with ovarian cancer for the past 20 years [38]. However, ∼80% of ovarian cancer cases are diagnosed at an advanced stage and these patients are intrinsically resistant to chemotherapy mainly due to ABCB1-mediated MDR [6, 7, 38]. Therefore, it is of vital medical significance to overcome or reverse ABCB1-mediated MDR in patients with ovarian cancer.

In the present study, afatinib robustly reversed the MDR to paclitaxel and adriamycin (Table 1), and increased the apoptosis ratio induced by paclitaxel in ABCB1-overexpressing cells (Figure 2). Knockdown of ABCB1 partly by siRNA further enhanced the reverse efficacy of afatinib on MDR (Table 2), indicating that the efficacy of afatinib in reversing MDR was associated with its interaction with ABCB1. More meaningfully, the combination of paclitaxel and afatinib induced significant tumor regressions (Figure 1F) and tumor necrosis (Figure 2C) of the A2780T xenografts *in vivo* as well as delaying their growth. It is reported that apatinib and saracatinib could reverse ABCB1-mediated MDR [13, 14, 17]. However, their efficacies were weaker than that of afatinib. In contrast, their concentrations needed for reversing MDR were higher. More importantly, they only delayed the growth of tumors, but not induced tumor regressions. Taken together, several different experiments suggest that afatinib has a superior efficacy in reversing ABCB1-mediated MDR both *in vitro* and *in vivo*.

Xiao-kun Wang et al. reported that afatinib could reverse ABCG2-mediated MDR, while not enhancing the cytotoxicity of doxorubicin in resistant cells that expressed ABCB1 [39]. By comparison, we found that there was a significant difference in the susceptibility of various cells to afatinib alone. Human ovarian cancer cell lines used in our study are much more resistant to afatinib than cells used in Xiao-kun Wang's study. Therefore, the reversal concentrations of afatinib used in ABCB1-overexpressing cell lines in our study (0.375–2.5 μM) were much higher than those used in his study (0.025–0.1 μM). Afatinib under 0.25 μM also couldn't reverse the drug-resistance to adriamycin and paclitaxel in this study. Therefore, the reversal effect of afatinib on MDR may be concentration dependent. The huge concentration differences of afatinib between the two studies may be responsible for the different results.

Mechanistically, afatinib significantly enhanced the intracellular accumulation of rhodamine 123 in ABCB1-overexpressing cells and tumors by decreasing the efflux function of ABCB1. In consideration of the universality of ABCB1 substrate drugs and the increasing use of afatinib in the clinic, these quantitative results offered a theoretical basis for dosage adjustments of ABCB1 substrate drugs during their combination treatment with afatinib in the clinic. Furthermore, several TKIs could interact with ABCB1, thereby inhibiting its efflux of chemotherapeutic agents [13, 17]. However, the mechanisms are still not very clear. Most reports hypothesize that these TKIs are also substrates of ABCB1 and share substrate binding domains with chemotherapeutic drugs. The competitive binding of these TKIs to the substrate binding domains relieves the binding of chemotherapeutic drugs, thereby decreasing their efflux [18]. In this study, afatinib could stimulate the ATPase activity of ABCB1 and was less cytotoxic in ABCB1-overexpressing cells, indicating that afatinib may also be a substrate of ABCB1. Further, by homology modeling and a docking simulation, we found that afatinib could be docked into a substrate binding domain of ABCB1. This finding also supports the hypothesis that afatinib could inhibit the efflux function of ABCB1 by competitively binding to the substrate binding domain.

In addition, we hypothesize that TKIs can inhibit the function of ABCB1 by binding to the ATP binding domain of ABCB1 and thereby cut the energy supply needed for transport process. No studies have tested this hypothesis. Similarly, by docking simulations, we found that afatinib might bind to the ATP binding domain of ABCB1 with high affinity by forming hydrogen bonds with Gly533, Gly534, Lys536 and Ala560 sites of ABCB1 (Figure 6C). Most ABCB1 inhibitors compete with other ABCB1 substrates for access to the binding pocket on ABCB1 and are called type I inhibitors. Their inhibitory effects are often limited as achieving an inhibitory effect on ABCB1 often requires high concentrations, which may cause toxicity problems. In fact, the first generation of ABCB1 inhibitors failed in the clinic mainly because of this problem. More effective and promising, type II inhibitors could bind to the ATP binding domains of ABCB1, cutting off the energy supply needed during the efflux process. Or they could bind to other domains that can modulate the conformation of ABCB1 and thus abolish the efflux function of ABCB1. Therefore, the finding that afatinib might bind to the ATP binding domain of ABCB1 could be the reason for the superior efficacy of afatinib in reversing ABCB1-mediated MDR. Furthermore, since substrate promiscuity is a hallmark of ABCB1 activity, a structural description of poly-specific drug-binding is important for the rational design of ABCB1 inhibitors [40]. From this viewpoint, the human ABCB1 model generated in this study, along with the docking simulation attempts may help subsequent researchers verify whether a drug has the potential to be developed as a reversal agent for ABCB1-mediated MDR.

The reversal of ABCB1-mediated MDR could be achieved either by inhibiting the function or attenuating the expression of ABCB1. Further studies showed that afatinib could attenuate the expression of ABCB1 both at protein and mRNA levels (Figures 4 and 5A). It is widely reported that TKIs could inhibit the function of ABCB1 [13–15]. However, to our best knowledge, it hasn't been reported that any TKI at nontoxic concentrations could attenuate the expression of ABCB1. In this study, lapatinib, a reversible TKI targeting EGFR and HER-2, also could not attenuate the expression of ABCB1. On the contrary, longtime administration of imatinib and lapatinib could enhance the expression of ABCB1 as they are substrates of ABCB1, which even aggravates the resistance of other combinational chemotherapy drugs [18, 19]. Herein, for the first time, we found that afatinib at nontoxic concentrations could dynamically reverse MDR by attenuating the expression of ABCB1, while reversible TKIs couldn't achieve that. This finding indicates that afatinib is a more promising and unique reversal agent for ABCB1-mediated MDR.

NF-κB is an important factor in the transcriptional regulation of many genes. Inactive NF-κB dimers, composed of p65 and p50 subunits, are sequestered in the cytoplasm in association with the inhibitory molecules of the IκB family. Stimulation on cells causes phosphorylation of the inhibitor IκB, leading to its polyubiquitination and proteasome-mediated degradation, and the release of active NF-κB. ABCB1 is encoded by the *MDR*1gene, which is located on 7, q21.1 chromosome. It is reported that an NF-κB binding domain (5′-GGGGAATTCC-3′) is located between base pairs −167 and −158 of *MDR*1 promoter region and could activate *MDR*1 gene transcription [41]. Furthermore, inhibiting the activation of the NF-κB could attenuate the expression of ABCB1 [42]. In this study, afatinib attenuated the expression and nuclear translocation of the NF-κB subunit p65 and blocked both constitutive and inducible activation of the NF-κB. In addition, both PDTC (a specific NF-κB inhibitor) and p65-specific siRNA attenuated the expression of ABCB1 (Figure 5A–5C). These results indicated that afatinib could attenuate the expression of ABCB1 transcriptionally by inhibiting the activation of NF-κB. Further studies showed that upstream PI3K/AKT and MAPK/p38 pathways were involved in the regulatory effect of afatinib on the activation of NF-κB. As inhibition of the PI3K/AKT pathway may partially restore sensitivity to cisplatinum in cancer cells [43], this finding also explains the reason why afatinib could reverse the resistance to cisplatinum, which is not a substrate of ABCB1 (Table 1). Considering that paclitaxel and platinum combination chemotherapy has remained the global first-line chemotherapy regiment for patients with ovarian cancer. This finding further proves the superiority of afatinib as a reversal drug. To sum up, afatinib attenuated the expression of ABCB1 by inhibiting its transcription via down-regulation of PI3K/AKT and MAPK/p38-dependent activation of NF-κB (Figure 5G).

Afatinib, an EGFR-targeted TKI, obtained FDA approval for first-line treatment in EGFR-mutated NSCLC patients. These mutations include exon 19 deletions and exon 21 (L858R) substitution mutations. It means that afatinib is more effective in EGFR-mutated cancer cells. Therefore, it's possible that the efficacy of afatinib in reversing ABCB1-mediated MDR in multidrug resistant cells is because of EGFR mutations occurring in multidrug resistant cells. To exclude this possibility, gene sequencing experiments were performed and the results showed that EGFR mutations were not correlated with the reversal efficacy of afatinib on MDR in ovarian cancer.

The most critical challenge for TKI therapeutics is also the development of drug-resistance [18]. Despite an initial response, patients almost invariably become resistant to TKIs gradually and relapse after several months. One important reason is that most approved TKIs are substrates and are transported by ABC transporters, resulting in altered pharmacokinetics or an obvious resistance to these drugs in cancer patients [19]. More seriously, as a substrate, longtime treatment often induces the up-regulation of ABC transporters, which further aggravates the resistance of conventional chemotherapy drugs [19, 20]. Consequently, TKIs that could attenuate the expression of ABC transporters may affect their own metabolism and thus diminish their own resistance. From this viewpoint, afatinib may overcome its own ABCB1-mediated drug resistance in the clinic. As expected, it was reported that afatinib showed a potent inhibitory activity even in *in vitro* models which were resistant to first-generation EGFR TKIs, and was still effective in NSCLC patients that showed no or low response to gefitinib or erlotinib [44]. We speculate that its covalent binding to tyrosine kinase active domains and its irreversible inhibition on EGFR and HER-2 may be responsible for these phenomena, as an irreversible tyrosine kinase blockade may result in a longer suppression of ErbB signaling, leading to better treatment outcomes. However, more studies should be taken, especially structural chemistry assays.

In conclusion, this study clearly demonstrates that afatinib is a potential and superior reversal drug for ABCB1-mediated MDR in human ovarian cancer. This study also strongly advocates for the combination treatment of afatinib and ABCB1 substrate chemotherapeutic drugs. Furthermore, considering the universality of ABCB1 substrate drugs and the increasing use of afatinib in the clinic, the quantitative results of this study also offer a theoretical basis for dosage adjustments of ABCB1 substrate drugs during their combination treatment with afatinib. Finally, this study can facilitate the rediscovery of superior MDR reversal agents from molecular targeted drugs to provide a more effective and safer way of resensitizing MDR.

## MATERIALS AND METHODS

### Reagents and antibodies

Afatinib, adriamycin and cisplatinum were purchased from Meilun Biology Technology Co., Ltd (Dalian, China). Paclitaxel was obtained from Tianfeng Technology Co., Ltd (Xi'an, China). Rhodamine 123, MTT, verapamil, lipopolysaccharide (LPS), PDTC, LY294002 and other chemicals were purchased from Sigma Chemical Co. (St. Louis, MQ, USA).

The monoclonal antibody against ABCB1 was obtained from Santa Cruz Biotechnology Inc. (CA, USA). Antibodies against iκBα, p-iκBα, p65 and Lamin B1 were purchased from Epitomics, Inc. (California, USA). Antibodies against EGFR, p-EGFR, HER-2, p-HER-2, AKT, p-AKT, p38, p-p38, ERK1/2, p-ERK1/2, JNK, p-JNK and GAPDH were obtained from Bioworld Technology, Inc. (Minnesota, USA). The HRP-conjugated goat anti-rabbit secondary antibody and the CY3-conjugated goat anti-rabbit secondary antibody were purchased from Abcam Inc. (Cambridge, MA, USA).

### Cell lines and cell culture

Human ovarian cancer cell line A2780 and its multidrug-resistant subline A2780/T were purchased from KeyGEN Biotech Co. (Nanjing, China). SKOV3 and its multidrug-resistant subline SKOV3-DDP cells, as well as normal human liver LO2 cells were kindly provided by doctor Li Zhang (Nanfang hospital). All cell lines were cultured in DMEM or RPMI 1640 medium, supplemented with 10% fetal bovine serum (Gibco, Grand Island, NY, USA). Additionally, the medium of A2780/T and SKOV3-DDP cells contained 800 ng/ml paclitaxel to maintain the activity of MDR and was changed to drug-free culture medium two weeks before assay.

### Cytotoxicity assay

Cytotoxicity was determined using the MTT assay. The IC_50_ was calculated from survival curves using the Bliss method [45]. The resistance degree was estimated through dividing the IC_50_ of the MDR cells by that of the parental sensitive cells. The fold-reversal factor of MDR was calculated through dividing the IC_50_ of the anticancer drug in the absence of reversal agents by that in the presence of reversal agents.

### Small interfering RNA assay

Synthetic small interfering RNA (siRNA) specific for human ABCB1 (ABCB1-siRNA) and NF-κB subunit p65 (p65-siRNA) were purchased from Suzhou GenePharma with the following sequences: 5′-GUGGGCACAAACCAGAUAATT-3′ (forward) and 5′-UUAUCUGGUUUGUGC CCACTT-3′ (reverse) for ABCB1-siRNA; 5′-CCUUUC UCAUCCCAUCUUU TT-3′ (forward) and 5′-AAAGAUGGGAUGAGAA AGGTT-3′ (reverse) for p65-siRNA-1; 5′-GGACAUA UGAGACCUUCAATT-3′ (forward) and 5′-UUGAAGG UCUCAUAUGUCCTT-3′ (reverse) for p65-siRNA-2. The transfecting process was conducted by using the lipofectamin 2000 transfection reagent (Carlsbad, CA, USA) and Opti-MEM (Carlsbad, CA, USA) according to the manufacturer's protocol.

### Mouse xenograft model

Athymic female nude mice (BALB/c-nu/nu) of 4∼6 weeks old were used to establish the A2780T xenograft model following the Declaration of Helsinki and the guidelines from Southern Medical University Institutional Animal Care and Use Committee. Briefly, 5 × 10^6^ A2780T cells were suspended in 200 μl PBS and inoculated subcutaneously into the flanks of the nude mice. When tumors reached a mean diameter of 0.6 cm, the mice were randomized into four groups (9 in each group) and received different treatments: (a) saline (q3d × 6, intraperitoneally [i.p.]); (b) paclitaxel (18 mg/kg, i.p., q3d × 6); (c) afatinib (70 mg/kg, i.p., q3d × 6); and (d) paclitaxel (18 mg/kg, i.p., q3d × 6) plus afatinib (70 mg/kg, i.p., q3d × 6). Throughout the treatment, the mice were weighed and their tumors were measured with a caliper every 3 days. Tumor volumes (V) were calculated using the formula: V = π/6 × larger diameter × (smaller diameter)^2^. When tumors grew to a proper size, the mice were euthanized and the tumors were excised, weighed and stored at −80°C or 4% paraformaldehyde for further examination. The ratio of growth inhibition (IR) was calculated according to the following formula:

$$\text{Inhibition rate}(\text{IR})=1-\frac{\text{mean tumor weight of treatment group}}{\text{mean tumor weight of control group}}\times 100\%$$

### Apoptosis assays

Cell apoptosis were measured by using the Dead Cell Apoptosis Detection Kit from Invitrogen (Carlsbad, CA, USA) according to the manufacturer's instructions. Then, the apoptosis rates of cells were detected by flow cytometry. TUNEL assay was performed using the DeadEnd™ Colorimetric TUNEL System from Promega (Madison WI, USA) following the manufacturer's instructions. Then, the apoptosis of tumor tissues was detected by a Zeiss LSM710 confocal laser microscope (Carl Zeiss, Jena, Germany).

### Rhodamine 123 accumulation assay

Effects of afatinib on the intracellular accumulation of rhodamine 123 were investigated. Briefly, 1 × 10^6^ cells were collected, centrifuged, washed once with PBS and re-suspended in 1.5 ml complete medium with 5 μg/ml rhodamine 123 in the absence or presence of various concentrations of afatinib. Subsequently, cells were incubated at 37°C for 2 h. After incubation, cells were centrifuged, washed three times with cold PBS and re-suspended in 0.5 ml cold PBS. The mean fluorescence intensity, reflecting the intracellular concentration of rhodamine 123, was determined by flow cytometric analysis subsequently. Furthermore, verapamil was used as a positive control.

### *In vivo* imaging assay

Athymic nude mice bearing A2780T cell xenografts were established following the method described in the *in vivo* xenograft model section. When tumors reached a mean diameter of 1.5 cm, the mice were randomized into three groups (3 in each group) and injected with different solutions into the vein of the tail: (a) saline (400 μl); (b) rhodamine 123 (20 mg/kg, 400 μl); and (c) afatinib + rhodamine 123 (afatinib, 4 mg/kg; rhodamine 123, 20 mg/kg; 400 μl). 90 minutes after injection, the mice were anesthetized with 0.3% pentobarbital sodium, placed in a prone position on a supporting cradle and moved into the scanner. Then, the whole body scans of the mice under the fluorescence transillumination model were collected by the In-Vivo Imaging System (Kodak Carestream Health, Rochester, NY, USA). The excitation wavelength and emission wavelength were set at 465 nm and 535 nm, respectively. The quantitative analysis of the results was accomplished by the Kodak Molecular Imaging (MI) Software.

### *In vitro* transport assay

Effects of afatinib on the efflux of rhodamine 123 were investigated. Briefly, 1 × 10^6^ cells were collected, centrifuged and washed once with PBS before being re-suspended in 1.5 ml complete medium supplemented with 5 μg/ml rhodamine 123. Subsequently, cells were incubated at 37°C for 2 hours. After incubation, cells were centrifuged, washed twice with cold PBS and re-suspended in complete medium in the absence or presence of different concentrations of afatinib. Then, the cells were incubated at 37°C for 30 minutes. In the control experiments, the same reactions were kept at 0°C. After incubation, the cells were centrifuged, washed three times with cold PBS and resuspended in 0.5 ml cold PBS for flow cytometric analysis. The quantity of the rhodamine 123 efflux by ABCB1 was calculated by subtracting the values obtained at 37°C from the values obtained at 0°C.

### ATPase activity assay of ABCB1

Effects of afatinib on the ATPase activity of ABCB1 were measured by using the P-gp-Glo™ Assay Systems from Promega (Madison WI, USA) according to the manufacturer's protocol. A detailed description was supplied in Supplementary Materials and Methods.

### RT-PCR and western blot

RT-PCR and western blot were performed as described previously [42], and as detailed in the Supplementary Materials and Methods section. The sequences of specific PCR primers were 5′-TGGGGCTGGACTTCCTCTCATGATGC-3′ (forward) and 5′-GCAGCAACCAGCACCCC AGCACCAAT-3′ (reverse) for *MDR*1; 5′-AGAAGGCTGGGGCTCAT TTG-3′ (forward) and 5′-AGGGGCCATCCACAGTC TTC-3′ (reverse) for GAPDH.

### Cell immunofluorescence

The nuclear translocation of the NF-κB subunit p65 was detected by cell immunofluorescence. Briefly, A2780T cells were seeded on cover slips in 24-well plates and induced with different treatments. Afterwards, the cells were fixed in 4% paraformaldehyde in PBS for 10 minutes, washed three times with PBS and then permeabilized with 0.25% Triton X-100 for 10 minutes. Fixed cells were saturated for 30 minutes in 5% BSA at room temperature. Subsequently, cells were stained with the primary antibody (p65, 1:200 dilution) overnight at 4°C, followed by an incubation with the secondary antibody conjugated with CY3. DAPI was used to visualize the nuclei. Fluorescence images were captured using a Fluoview FV10i confocal microscope (Olympus, Center Valley, PA, USA).

### Immunohistochemistry and immunofluorescence

Immunohistochemistry and immunofluorescence were performed as described previously [46]. A detailed description was supplied in Supplementary Materials and Methods.

### Pharmacophore model

To vertify the structural basis of afatinib as an ABCB1 inhibitor, a novel pharmacophore of ABCB1 inhibitors was generated. Briefly, the common features for afatinib, tariquidar, and zosuquidar were generated with a pharmacophore protocol using the Discovery Studio 3.5 [33, 34]. Six features including the hydrogen bond acceptor (HBA), hydrogen bond donor (HBD), hydrophobic center (HP), positive charge center (POS), negative charge center (NEG), and aromatic ring (AR) were selected. The number of each feature was set to range from 0 to 5. The top ten pharmacophores were generated with one being selected on the basis of the best match between afatinib and the pharmacophore model.

### Protein structure preparation and docking simulation

The X-ray crystal structure of mouse ABCB1 (PDB ID: 3G61), obtained from the RCSB Protein Data Bank, was used as the template to build the homology model of human ABCB1 [40]. The sequence alignment between mouse ABCB1 and human ABCB1 (Supplementary Figure S5) was performed to assist the model amendment process. The initial homology model was constructed in Molecular Operating Environment (MOE 2013) and further optimized using AMBER 11 [47]. Various validation methods, including PROCHECK, ERRAT and PROSA were employed to evaluate the reliability of the human ABCB1 model [48, 49]. The substrate binding domain and the ATP binding domain were identified using Site Finder module in MOE 2013. The 3D structure of afatinib was built in MOE and energy minimized using the CHARMM force field. Different protonation states of afatinib with pH values ranging from 6.5 to 8.5 and all possible tautomers were generated. Each conformation of afatinib was docked. Docking protocol in MOE was employed to carry out the docking study.

### EGFR and ABCB1 mutational analysis

DNA was extracted from cells using the Tissue DNA Kit (OMEGA; Norcross, GA) according to the manufacturer's protocol [50]. PCR primers were designed to amplify a 405-bp fragment encompassing the sequence for exon 18 of EGFR gene, a 469-bp fragment encompassing the sequence for exon 19 of EGFR gene, a 384-bp fragment encompassing the sequence for exon 20 of EGFR gene, a 379-bp fragment encompassing the sequence for exon 21 of EGFR gene, a 378-bp fragment of *MDR*1 gene encompassing the sequence for Gly533, Gly534, Lys536 and Ala560 of ABCB1 protein, respectively. The primers were listed below. DNA sequencing was performed by an ABI 3730XL Auto-mated sequencer, using the ABI Prism Dye Terminator Cycle Sequencing Ready Reaction Kit (Perkin Elmer, Branchburg, NJ).

Exon 18-F 5′-CTTCCAAATGAGCTGGCAAGTG-3′
Exon 18-R 5′-GGAGTTTCCCAAACACTCAGTG-3′
Exon 19-F 5′-GGTGCATCGCTGGTAACAT-3′
Exon 19-R 5′-TCACTCATCATGCGTGTCAA-3′
Exon 20-F 5′-GATCGCATTCATGCGTCTTCAC-3′
Exon 20-R 5′-CACATATCCCCATGGCAAACTC-3′
Exon 21-F 5′-CGTTCGCCAGCCATAAGT-3′
Exon 21-R 5′-TCCTCCCCTGCATGTGTTAA-3′
MDR1-F 5′-CTTGGGCTGTGTATAGGATTCC-3′
MDR1-R 5′-GGAATCACCTAGAAGCTATCAG-3′

### Statistical analysis

Data were represented as the mean ± standard deviation and analyzed pairwise for statistical significance using *Student's t* test. For all tests, *p* < 0.05 was considered as statistically significant.

## SUPPLEMENTARY FIGURES AND TABLES


