# Supplementary material for: Afatinib reverses multidrug resistance in ovarian cancer via dually inhibiting ATP binding cassette subfamily B member 1
Source: Oncotarget. 2015 Jul 20;6(28):26142–60. doi: 10.18632/oncotarget.4536 (PMC4694892; doi:10.18632/oncotarget.4536)
Supplement: Supplementary file 1 [file oncotarget-06-26142-s001.pdf]

SUPPLEMENTARY MATERIALS AND METHODS

ATPase activity assay of ABCB1

Effects of afatinib on the ATPase activity of ABCB1 were measured by using the P-gp-Glo™ Assay Systems from Promega (Madison WI, USA) according to the manufacturer's protocol. Briefly, 20 μl of diluted human ABCB1 membranes (1.25 mg/ml) were added to each well containing different concentrations of afatinib or control drugs (0.1 mM Na_3VO_4 and 0.2 mM verapamil were respectively used as negative control and positive control drugs). After incubation at 37°C for about 5 minutes, reactions were initiated by adding 10 μl of 25 mM MgATP. Then, the plate was mixed on a plate shaker for 40 minutes at 37°C. After that, the plate was removed from the 37°C heat source and luminescence was initiated by adding 50 μl of ATP detection reagent. Subsequently, the plate was incubated at room temperature for 20 minutes to allow the luminescent signal to develop. Finally, the luminescence of each well was read on a SpectraMax M5 microplate reader (Molecular Devices, Sunnyvale, CA, USA). To quantitatively investigate the effect of afatinib on the consumption of ATP, MgATP was used for establishing a standard curve to convert relative light unit (RLU) values into ATP concentrations. The calculation formula is as follows:

$$\text{n mol ATP consumed/}\mu\text{g ABCB1/minute} = \frac{[\text{ATP}(Na_3VO_4)] - [\text{ATP(TC)}]}{(25\ \mu\text{g ABCB1} \times 40\ \text{minutes})}$$

RT-PCR

Total RNA was extracted with Trizol and reverse-transcribed to complementary cDNA using the PrimeScript™ RT reagent Kit (Takara, Shiga, Japan) according to the manufacturer's instructions. RT-PCR was performed using the SYBR Premix Ex Taq Kit (Takara, Shiga, Japan) and the ABI Prism 7500 Sequence Detector System (Applied Biosystems, Foster City, CA, USA).

Western blot

Protein extracts were obtained by lysing cells using the Total Protein Extraction Kit (KeyGen Biotech, Nanjing, China). Nuclear and cytosolic extracts were obtained using KeyGen Nuclear and Cytoplasmic Protein Extraction Kit (KeyGen Biotech, Nanjing, China). Protein concentrations were quantified using the BCA Protein Assay Kit (Beyotime, Shanghai, China). Equal amounts of protein (20 μg) were resolved by SDS-PAGE on a 10% polyacrylamide gel and transferred onto a polyvinylidene fluoride membrane. After saturation in 7.5% nonfat milk at room temperature for 1 hour, the membranes were incubated overnight in the corresponding primary antibodies at 4°C. Then, bound antibodies onto membranes were detected using horseradish peroxidase-conjugated secondary antibodies, followed by the enhanced chemiluminescence detection reagents (Millipore, MA, USA).

Immunohistochemistry

Briefly, tissue slides were deparaffinized and rehydrated. Antigen retrieval was carried out by heating in a solution of 0.01 M citrate buffer (pH = 6.0) twice for 5 minutes. The tissue chips were incubated with 3% (v/v) H_2O_2 at room temperature for 10 minutes, rinsed twice and blocked in 10% normal goat serum for 30 minutes. The slides were then incubated overnight with the anti-ABCB1 monoclonal antibody (1:100 dilution) at 4°C, followed by an incubation with the secondary antibody for 30 minutes at room temperature. After rinsing in PBS three times, all slides were visualized with 0.05% (w/v) 3, 3′-diaminobenzidine as the chromogen and then counterstained with hematoxylin.

SUPPLEMENTARY FIGURES AND TABLE

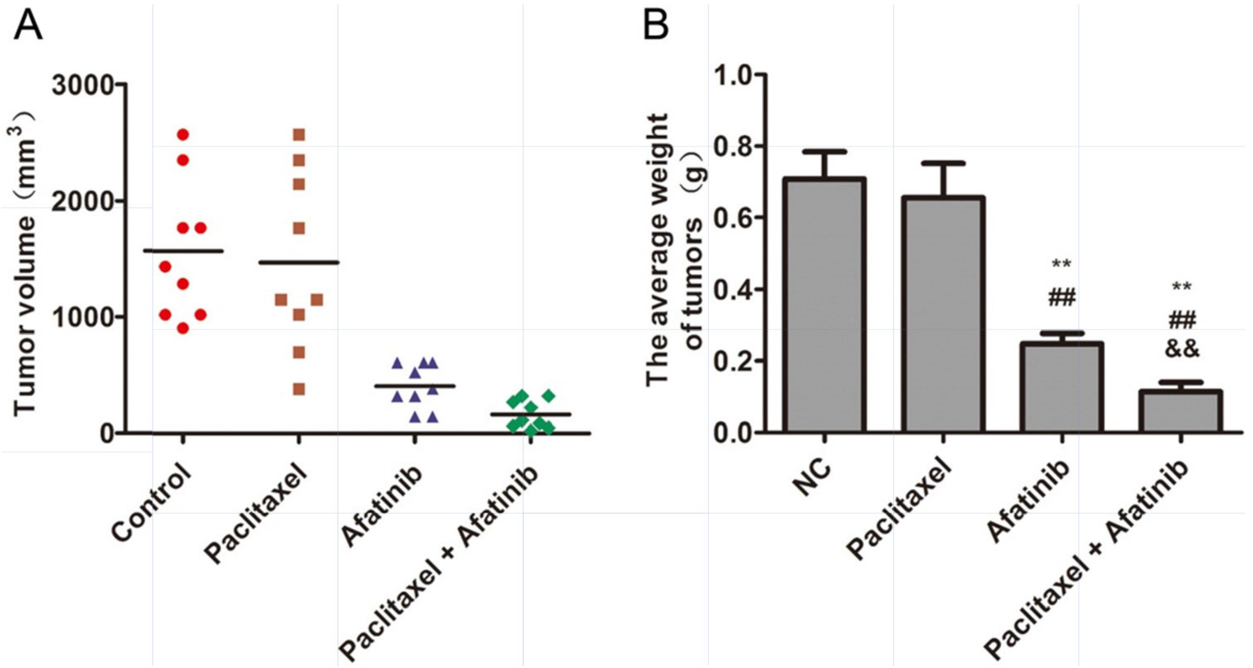

Supplementary Figure S1: The volume distributions and the average weights of tumors in four groups. A. The volume distributions of tumors in four groups. **B.** The average weights of tumors in four groups. Shown are the mean weights of tumors in each group with standard deviations. ($n = 9$); $^{**}p < 0.01$ vs control group; $^{\#\#}p < 0.01$ vs paclitaxel group; $^{\&\&}p < 0.01$ vs afatinib group.

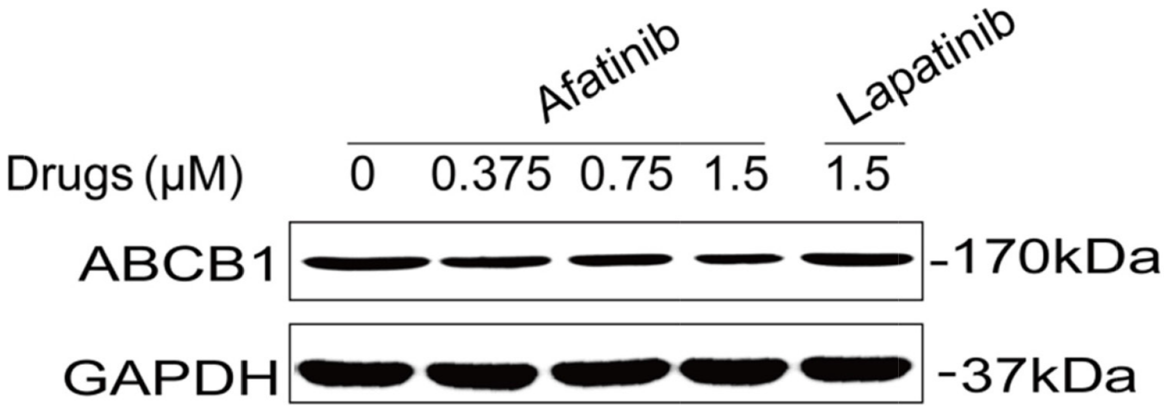

Supplementary Figure S2: Afatinib attenuated the expression of ABCB1 protein in SKOV3-DDP cells. SKOV3-DDP cells were treated with indicated concentrations of afatinib or lapatinib for 48 hours. Subsequently, the lysates were subjected to western blot analysis.

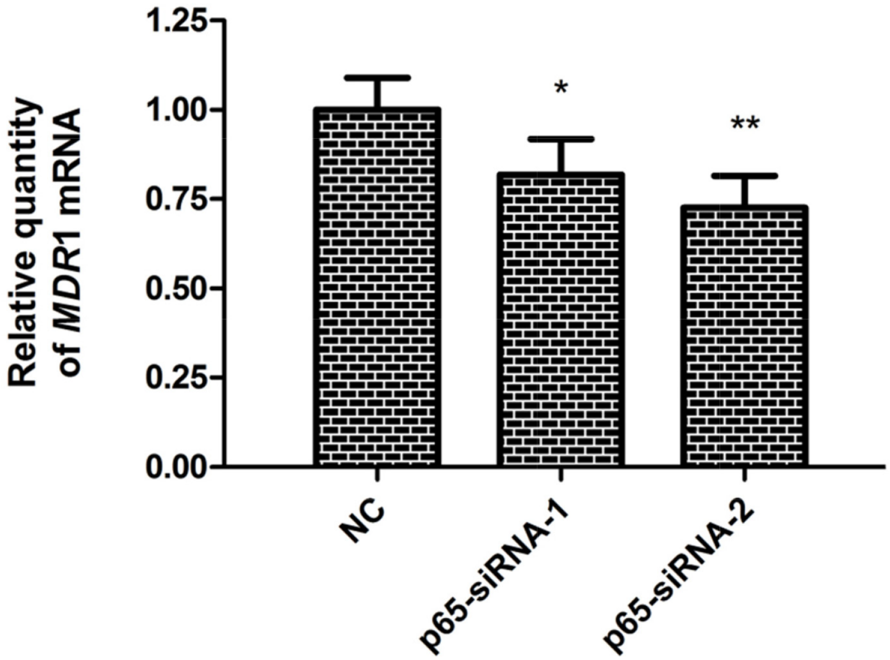

Supplementary Figure S3: Knocking down the expression of NF-κB subunit p65 significantly attenuated the expression of *MDR1* mRNA in multidrug resistant A2780T cells. $^{*}p < 0.05$ vs control group; $^{**}p < 0.01$ vs control group.

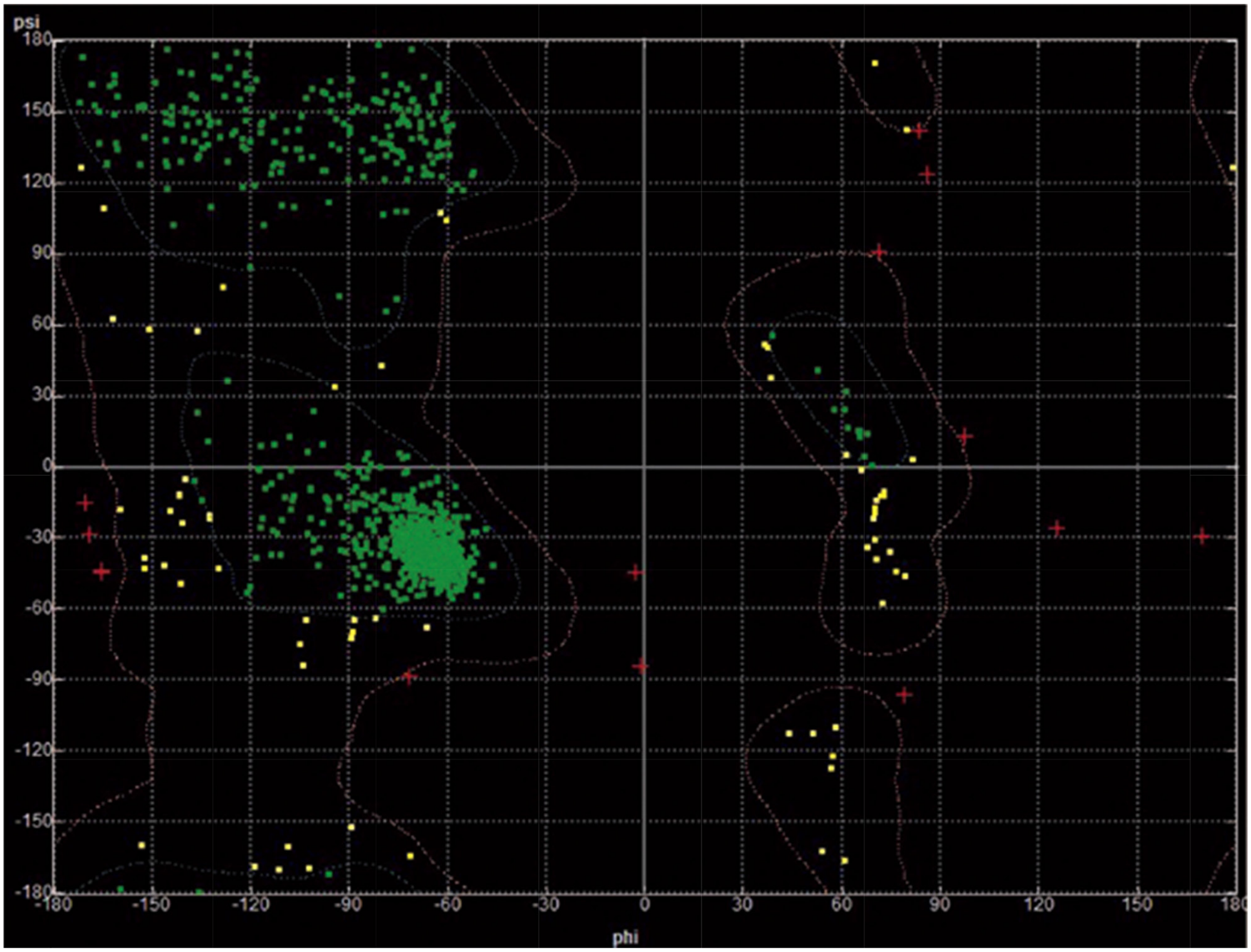

Supplementary Figure S4: The ramachandran plot for the human ABCB1 model. The core, allowed and outlier residues are shown in green, yellow and red respectively.

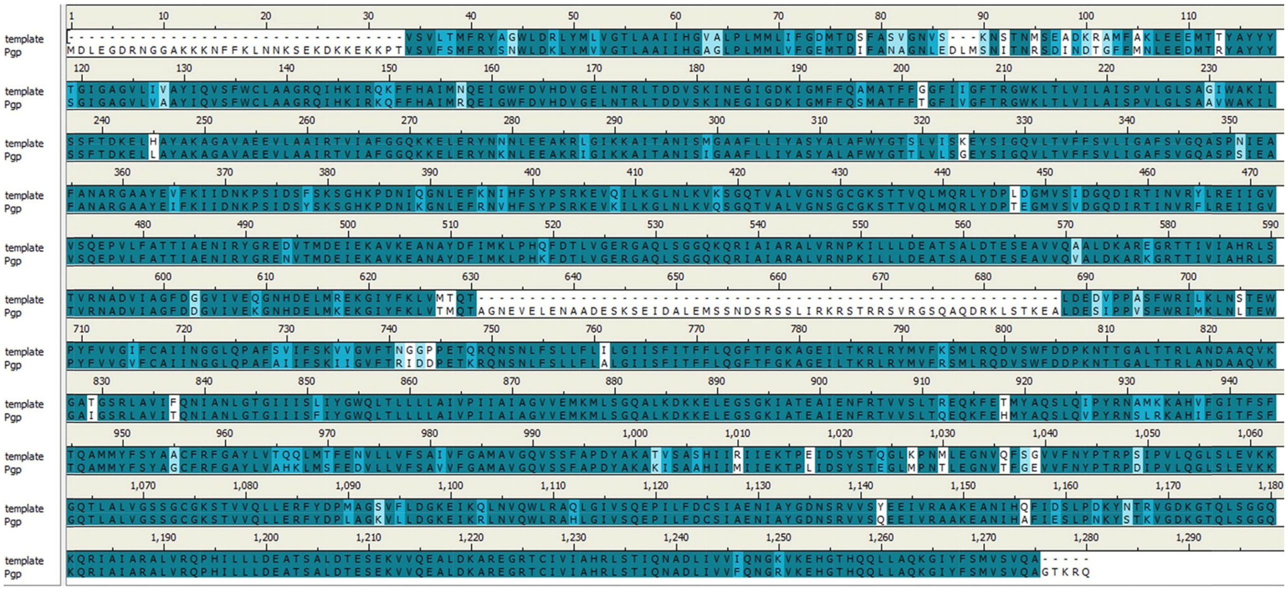

Supplementary Figure S5: Sequence alignment of human ABCB1 protein (below) and mouse ABCB1 protein (above) were conducted to adjust the generated human ABCB1 homology model. There were high level homologies between human ABCB1 protein and mouse ABCB1 protein, indicating that the X-ray crystal structure of mouse ABCB1 could be used as the template to build the homology model of human ABCB1 protein.

Supplementary Table S1: Structural assessment of the generated human ABCB1 homology model

Models	Protein evaluation methods		
	PROCHECK[a]	ERRAT[b]	PROSA[c]
Constructed model	96.86%	89.88	–13.13
Optimized model	98.79%	96.76	–13.17
Template(PDB:3G61)	91.46%	74.68	–13.27

[a] PROCHECK was used to check the geometrical and stereochemical quality of the model.
[b] ERRAT was used to check the non-bonded atomic interactions.
[c] ProSA was applied to check the energy criteria of the model.
